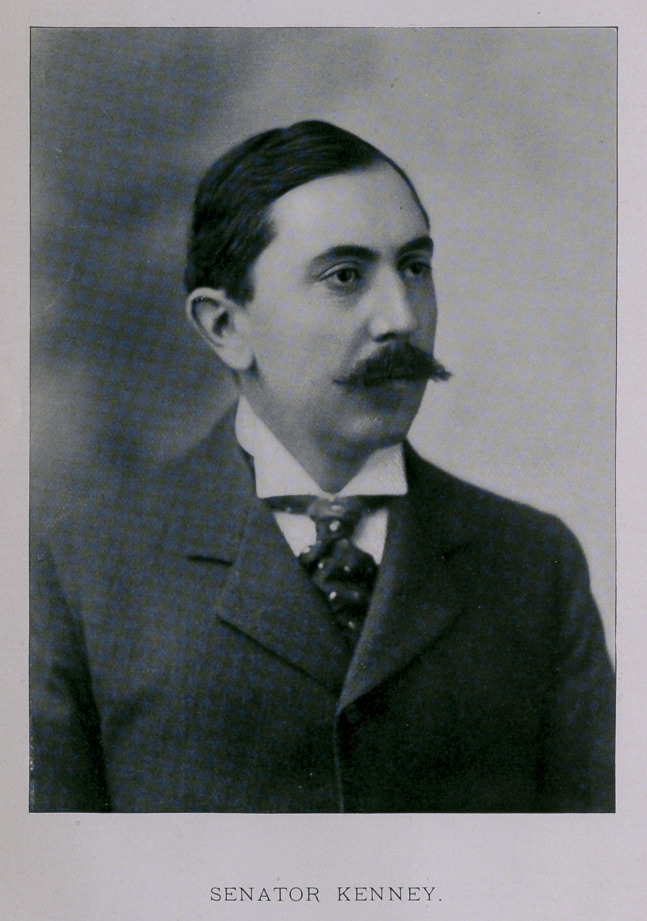# Richard R. Kenney, U. S. Senator from Delaware

**Published:** 1900-09

**Authors:** R. S. Huidekoper


SENATOR KENNEY.

### THE JOURNAL OF COMPARATIVE MEDICINE AND VETERINARY ARCHIVES.

VOL. XXI. *SEPTEMBER, 1900.* No. 9.

### RICHARD R. KENNEY,

U. S. Senator from Delaware.

REMARKS BY R. S. HUIDEKOPER.[1]

IT is a curious fact that several of the most important events in the foundation and development of veterinary medicine have been due to the interest and activity of soldiers, statesmen, and lawyers. Xenophon was the first to write of the care of the horse, especially its feet, of which he makes an important chapter in his *Anabasis*. Carlo Ruini, Senator of Bologna, who was one of the most advanced political leaders of Italy the latter half of the fifteenth century, wrote the first great book on the anatomy of the horse,[1] started the veterinary museum and library at the University of Bologna, now the largest and most complete in the world, and originated teaching on veterinary subjects.

Bourgelat, a French advocate, conceived the idea of and founded the first regular veterinary school—that at Lyons, in France—in 1762, and so interested the king and his mistress, Madame de Pompadour, in his work that he was called to Paris, given the old chateau with the menagerie near Vincennes, and founded the second school—that at Alfort—in 1765.

Here, within five years, came military officers sent by their governments in Austria, Prussia, and Denmark, to acquire the knowledge which was gaining such a reputation for its service to the army.

[1] Extract from the Proceedings of the Thirty-seventh Annual Meeting of the American Veterinary Medical Association, Detroit, Mich., September, 1900.

[2] First edition, 1592, in folio, profusely illustrated with full-page wood engravings, richly ornamented. This work describes the valves of the heart and clearly indicated the circulation of the blood, thirty years before the time Harvey obtains credit for the discovery.

A few years ago we were called upon to mourn the death of the Marquis Ercolani, scientist, statesmen, the friend of Victor Emanuel and Garibaldi, who, after completing the task of the consolidation of Italy, ended his days in the laboratories and library of the Veterinary School of the University of Bologna, continuing his researches in comparative medicine, and completing the library founded by Ruini until it held, at the time of his death, a copy of every book which had been published on veterinary medicines.

Virchow and Paul Bert, statesmen and scientists, were ardent veterinary students.[1]

Dr. Benjamin Rush, statesman, patriot, and surgeon of American Revolutionary days, first proposed a veterinary school in America.[2]

In 1855 General George B. McClellan, then a captain of the United States Army, was sent to Europe to report on the European armies. In his report he gave a long description of the veterinary schools of Berlin and Vienna, and recommended that "the general features of their system might be followed in our service with great advantage."

General Sheridan's untimely death prevented the organization of the veterinary service in our army which he had in view.

It remained for Senator Kenney, of Delaware, to appreciate the public good which would come to the United States Government, its army and people, by such a service, and to devote the time, enthusiasm, and energy requisite for its accomplishment. He undertook the work at the end of the third session of the Fifty-fifth Congress, and, as we all know, succeeded in passing his bill in the Senate for the organization of a veterinary corps in the army in the first session of the Fifty-sixth Congress.[3]

[1] Virchow, who was always the enthusiastic friend of the veterinarian, on a visit to Paris in the late '60s, found Paul Bert in his laboratory working over some physiological subject, and said: "My young friend, never let politics take you from your scientific work; it demands too much time." Some years after the Franco-Prussian War Paul Bert remarked, one morning at 7 o'clock, when I found him sitting at his desk in his shirt-sleeves, with a roll of bread and a glass of red wine and water for his breakfast, and his ante-room filled with politicians and students waiting for audience and advice, "And if Virchow had let politics alone in Germany I need not have taken them up in France." (Personal incident of the writer's.)

[2] In 1806 he recommended the establishment of such a school, on the models of those he had seen in Europe, at the University of Pennsylvania.

[3] Senator Kenney's bill for the establishment of an organized veterinary corps in the United States Army passed the United States Senate as "Section 14 of S. 4300," a bill for the efficiency of the army (known as the Army Organization Bill).

This Army Bill was under consideration for some ten days by the Military Committee of the House of Representatives, but action upon it has been delayed until the reconvening of Congress in December next.

Senator Richard Roland Kenney was born in Sussex County, Delaware, September 9, 1856. He was graduated from the Laurel Academy, Delaware, in June, 1874, and then attended Hobart College, Geneva, New York. He read law under the tuition of the late Senator Willard Saulsbury at Dover, and was admitted to the Bar October 19, 1881, where he has practised law since that time. He was elected State Librarian of Delaware in January, 1879, and held that office for two terms. In 1880 he organized the First Regiment, National Guards of Delaware, and served in it as Captain, Company D, for seven years, when he was appointed Adjutant General of the State by Governor B. T. Biggs. He occupied this position for four years until January, 1891.

He was a delegate to the National Democratic Convention at Chicago in 1892, and in 1896 was appointed a member of the National Democratic Committee. He was again a delegate to the National Convention at Kansas City in 1900, and was chosen to succeed himself as a member of the National Committee. Senator Kenney was elected to the United States Senate January 19, 1897, to fill the vacancy caused by the Legislature of 1895 failing to elect a Senator to succeed the Hon. Anthony Higgins, whose term expired March 4, 1895. He took his seat February 5, 1897. This term of his service will expire March 3, 1901.

Senator Kenney, while a Democrat in politics, is broad-minded and unbiassed on all questions of national importance and in progressive legislation for the public good. He voted for the ratification of the treaty of peace with Spain, and has made most liberal speeches in the Senate concerning our new possessions, Puerto Rico and the Philippines.

He made a brilliant and logical speech on the legal aspects of the case of the appointment of Senator Quay from Pennsylvania, and voted for seating him. He advocated ardently and reported favorably from the Senate Post-Office Committee the bill to classify the Post-Office clerks. He secured with great labor the appropriations and works for the river and harbor improvements in the State of Delaware.

Senator Kenney is an indefatigable worker, and never neglects the smallest details, nor considers any of the trivial favors for which he is constantly called upon too small to look after and attend to. As a secretary in his office remarked, on clearing up the desks on the last day of Congress, "Well, we have passed every pension bill we had, and have not a bill unaccomplished which has not been unavoidably delayed in some one else's office."

Such is a brief sketch of Senator Kenney, in whose hands is the guidance of our army legislation. He combines the ability and the will to accomplish what has been done, and we owe to him a debt of gratitude. Those who know legislation in Washington know what a task it was.

We also owe sincere thanks to Senator Wolcott, of Colorado; Senators Frye and Hale, of Maine; Senator Gallinger, of New Hampshire; and to Senator Stewart, of Nevada, all of whom took active personal interest in the support of our bill.[1]

Dr. Hoskins addressed the chair.

Mr. President and Gentlemen: I know of no occasion in the history of American veterinary medicine when our profession has created so deep an obligation to any one person for valuable services rendered, or when we have owed so great a debt of gratitude as we do to-day to Senator Richard R. Kenney, of Delaware, who has so ably led our battle in army legislation for rank and recognition for our profession in this branch of our government; and I am in thorough accord with the sentiment so well expressed by Dr. Huidekoper in his record of the services rendered. I desire to propose a vote of thanks from this Association to Mr. Kenney, and offer that our Secretary be instructed to convey the same to him under the official signature of our officers.

This motion was seconded and unanimously adopted.

On motion of Dr. Austin Peters, of Boston, the following resolution was unanimously passed:

*Resolved*, That we offer our sincere and cordial thanks to Senator Wolcott, of Colorado, and the other Senators who so kindly took an interest in securing the passage of our veterinary bill.

[1] The brilliant and forcible speeches of Senator Wolcott and others will be found in the Congressional Record of May 4, 1900.

The following Senators voted for the Veterinary Bill, though there were some fifteen more Senators who had promised their support, but were absent when the roll was called:

| | | | |
|---|---|---|---|
| Allison, | Frye, | McCumber, | Taliaferro, |
| Bacon, | Gallinger, | McEnery, | Teller, |
| Baker, | Gear, | Morgan, | Turner, |
| Chandler, | Hale, | Nelson, | Wolcott. |
| Clay, | Hansbrough, | Perkins, | |
| Culberson, | Kenney, | Quarles, | |
| Foster, | McComas, | Stewart, | |

## Figures and Tables

**Figure f1:**